# A database for G proteins and their interaction with GPCRs

**DOI:** 10.1186/1471-2105-5-208

**Published:** 2004-12-24

**Authors:** Antigoni L Elefsinioti, Pantelis G Bagos, Ioannis C Spyropoulos, Stavros J Hamodrakas

**Affiliations:** 1 Department of Cell Biology and Biophysics, Faculty of Biology, University of Athens, Athens 157 01, Greece

## Abstract

**Background:**

G protein-coupled receptors (GPCRs) transduce signals from extracellular space into the cell, through their interaction with G proteins, which act as switches forming hetero-trimers composed of different subunits (α,β,γ). The α subunit of the G protein is responsible for the recognition of a given GPCR. Whereas specialised resources for GPCRs, and other groups of receptors, are already available, currently, there is no publicly available database focusing on G Proteins and containing information about their coupling specificity with their respective receptors.

**Description:**

gpDB is a publicly accessible G proteins/GPCRs relational database. Including species homologs, the database contains detailed information for 418 G protein monomers (272 Gα, 87 Gβ and 59 Gγ) and 2782 GPCRs sequences belonging to families with known coupling to G proteins. The GPCRs and the G proteins are classified according to a hierarchy of different classes, families and sub-families, based on extensive literature searchs. The main innovation besides the classification of both G proteins and GPCRs is the relational model of the database, describing the known coupling specificity of the GPCRs to their respective α subunit of G proteins, a unique feature not available in any other database. There is full sequence information with cross-references to publicly available databases, references to the literature concerning the coupling specificity and the dimerization of GPCRs and the user may submit advanced queries for text search. Furthermore, we provide a pattern search tool, an interface for running BLAST against the database and interconnectivity with PRED-TMR, PRED-GPCR and TMRPres2D.

**Conclusions:**

The database will be very useful, for both experimentalists and bioinformaticians, for the study of G protein/GPCR interactions and for future development of predictive algorithms. It is available for academics, via a web browser at the URL:

## Background

G protein-coupled receptors (GPCRs), form one of the major groups of receptors in eukaryotes; they possess seven transmembrane α-helical domains, as confirmed by analysis of the crystal structure of Rhodopsin [1]. The study of GPCRs, and the way that they are activated by their ligands, is of great importance in current research aiming at the design of new drugs [2,3]. The importance of GPCRs in pharmaceutical industry, is reflected in the fact, that an estimated 50% of current prescription drugs target GPCRs [4-6]. Characteristically, the human genome, possesses approximately 700–800 GPCRs [7]. Understanding and studying the molecular mechanisms, through which the GPCRs transduce their signal into the cell, could also be an issue of great importance. There is a strong and accumulated body of evidence indicating that many GPCRs, form hetero-, or homo-dimers in order to transduce their signal [8].

Agonist binding to GPCRs leads to association of the hetero-trimeric G protein with the receptor, GDP-GTP exchange in the G protein α subunit followed by dissociation of the G protein into α-GTP and βγ complexes. The dissociated subunits can activate or inhibit several effector proteins such as adenylyl cyclase 1–9, PLCβ 1–4, tyrosine kinases, phosphodiesterases, phosphoinositide 3-kinase, GPCR kinases, ion channels, and molecules of the mitogen-activated protein kinase pathway, resulting in a variety of cellular functions [9]. However, there is evidence that some GPCRs transduce their signal through in a way that is not G protein-dependent [10], and also that hetero-trimeric G proteins are involved in mediating the action of some single-spanning membrane receptors [11]. Furthermore, some GPCRs have been shown to transduce signals into cells by coupling to small G proteins such as ADP ribosylation factor (Arf) and the dimeric Gh protein [10]. However, in the rest of this paper we will use the term G proteins to refer to hetero-trimeric G proteins, in order to avoid confusion, concerning the subunit composition of the trimers.

As mentioned above, G proteins, form hetero-trimers composed of Gα, Gβ and Gγ subunits. G protein α subunits, possess an intrinsic GTPase activity, which enables them to act as time switches: Hydrolysis of the bound GTP to GDP promotes the re-association of the α subunit with the βγ dimer and renders the G protein in an inactive form [12-14]. G protein trimers, are named after their α-subunits, which on the basis of their amino acid similarity and function are grouped mainly into four families [15]. These include, Gαs and Gαi/o, which stimulate and inhibit respectively an adenylate cyclase [16,17], Gαq/11 which stimulates a phospholipase C [18], and the less characterized Gα12/13 family that activates the Na+/H+ exchanger pathway [19]. At least 16 discrete subtypes of α subunits have been identified and classified into the above-mentioned families [20]. GPCRs, interact specifically with the α subunits of the G proteins, through their intracellular domains, however the same G protein may be activated by several receptors and the same receptor may couple to different G proteins, under different circumstances [15]. It is interesting to note, that not the whole intracellular loops of GPCRs, but rather the cytoplasmic extensions of the transmembrane helices, are directly involved in the interaction between G protein and GPCRs, as reported in studies involving site-directed mutagenesis and chimeric receptors [10,15]. This is confirmed in part, by a computational study, aiming at finding specific regular expression patterns that discriminate GPCRs with different coupling specificity [21].

Today, there exist general-purpose databases gathering information for receptors [22], and others, more specialised, focusing on GPCRs [23], and receptors of other types i.e. tyrosine kinase receptors [24], or ligand gated anion channels [25], but not a database focusing on the coupling specificity of the G proteins to their respective receptors. We have constructed a database, gpDB, built on a sophisticated relational scheme focusing on the coupling specificity of the α subunits of G proteins to their respective receptors. Such a database will be a complement to the already existing databases, and will be a useful tool for the study of the coupling specificity and the interaction of G proteins with GPCRs. Furthermore, the data collected in the database will be useful in the design of algorithms predicting the coupling specificity, and may provide useful insight towards understanding several aspects of protein-protein interactions.

## Construction and content

### Datasets

In order to construct the database, initial sequence information was retrieved from the publicly available databases: PIR [26], SWISS-PROT and TrEMBL [27]. In particular, a total of 418 entries for G proteins were retrieved, while, at the same time, we also retrieved 2782 GPCRs sequences with known coupling preference from SWISS-PROT/TrEMBL. The entries were obtained using suitable scripts written in Perl, in order to parse the DE (description) or the TITLE field in a SWISS-PROT or a PIR entry respectively. The datasets were then checked in order to eliminate duplicates. GPCRs sequences were obtained by using the keyword "G protein coupled receptor" and excluding those that were present in viruses. After the completion of the Uniprot database [28], all entries were checked again, and now we provide links solely to Uniprot (see below). Additional sequences that were not identified with the above-mentioned procedure were obtained manually after literature search. We used user-written Perl scripts to manipulate the data, whereas the annotations regarding: G Protein coupling specificity and effectors, GPCR dimerization and accessory proteins, and the corresponding references were appended manually in a spreadsheet. Regarding the GPCR/G protein interaction, the data was collected after an exhaustive and detailed literature search, mainly, following the classification of TiPS [29], and also [15] and references therein. At this point, we may emphasise, that the database does not report the potential coupling preference of a G protein to a GPCR, but only the naturally occurring coupling specificity. For instance, opsins that normally couple to Gαt (transducin) are expected to be able to functionally couple also to other members of the Gαi/o family. Since these Gα proteins, are not expressed in the same tissues as photoreceptors do, such a coupling is not reported. However, since there are also a lot of GPCRs, showing promiscuous coupling preference in heterologous expression systems [30,31], we could not fully discriminate cases of falsely reported coupling. This could be done, perhaps in a later version, when accumulated evidence of tissue expression patterns of GPCRs could be appended to the database. For G Protein/GPCRs coupling specificity, we provide links to PUBMED corresponding to original articles reporting the coupling preference observed in heterologous expression systems. We also provide links to published original articles, providing information about the dimerization status of a GPCR, and similar links for G Protein effectors and GPCRs accessory proteins.

### Implementation

**Figure 1:**
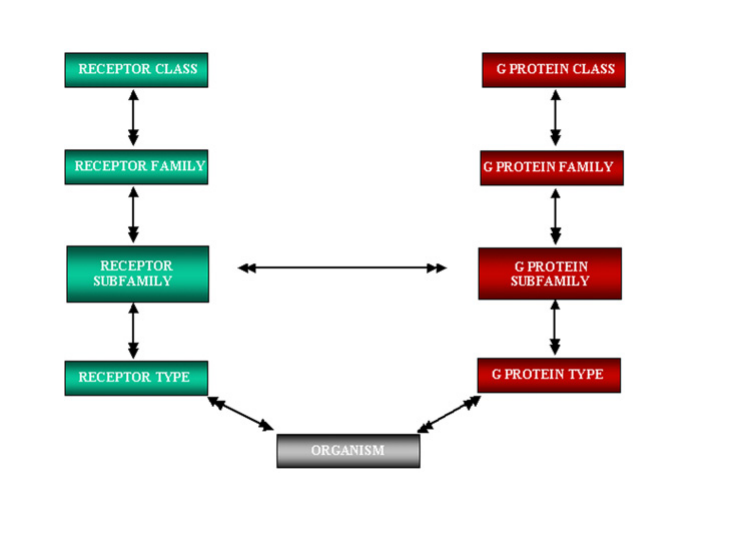
**The Relational Scheme of the database **Single-edged arrows indicate a "one-to-one" relation, whereas double-edged arrows indicate "one-to-many" relation. When there are double-edged arrows in both ends of a connection (for instance the Receptor subfamily – G protein type, interaction), this is an indication of relation of the type "many-to-many", i.e. a particular G protein may couple to receptors of different subfamilies, whereas a single receptor may couple to G proteins of different types.

The data has been organized on the basis of a relational model and is stored in a PostgreSQL database system. The user has supervisory access through our Apache web-server. The database is managed by interferential software, written in Java, which tends to settle any web-server's query. The main innovation of the database, resides on its relational scheme (Figure 1). It is well known, that the coupling specificity of G proteins to GPCRs, is not a one-to-one function. Thus, a particular GPCR, may couple to more than one G protein (promiscuous coupling), and vice-versa, one single G protein may couple to several GPCRs of the same organism, which is usually the case, considering the large number of different GPCRs and the much fewer types of G proteins. We have to mention here, that biologically functional complexes, involve trimers of G Proteins [10,20] and in many cases dimers of GPCRs [8,32], whereas there is also a variety of other molecules that could potentially interact with them, such as accessory proteins, scaffolds, and effectors [10]. Also, there is evidence, that there also exist single-spanning membrane receptors, whose actions are mediated by G Proteins [11]. However, even though we provide information on these interactions (where available), we did not attempt to organize the database in such a more complicated scheme, for several reasons. Firstly, there is not reported information in the literature for the majority of biological active G Protein heterotrimers, and the role that might play the trimer's different composition regarding subunits Gβ and Gγ. Secondly, even though there is a lot of evidence supporting the idea that GPCRs act as homo-, or hetero-dimers (evidence that the database is pointing to) [8,32,33], we could not provide a general scheme involving dimeric GPCRs activation, until more evidence will emerge, without the risk to fall in inaccuracies for the majority of receptors. Such features could be available in later versions of the database.

### Entry description – detailed view of an entry

Each database entry contains the following fields: gpDB name, gpDB id, UniProt accession number, Protein description and classification, sequence, species, organism common name, taxonomy, links to other databases (such as PDB, InterPro, Prints, Prosite, Pfam, GPCRDB, MIM or Smart) and coupling preference (if existent). Information on coupling preference is accompanied by links to PUBMED, corresponding to original articles reporting the interaction. There is also a field showing the reported effector molecules on which G Proteins act, and GPCRs accessory proteins, also accompanied by links to original articles. As we already noted, G proteins are classified into three classes (Gα, Gβ, Gγ). Gα class is further subdivided into four families (Gi/o, Gq/11, Gs, G12/13) and each family is subdivided into different subfamilies and types. This classification is mainly based on proteins present in vertebrates and in the vast majority of invertebrates, while some invertebrates (*C. elegans*) and all plants and fungi do not have such a detailed classification. Gβ and Gγ are subdivided into 6 and 13 different types, respectively. GPCRs are usually classified into several classes, according to the sequence similarity shared by the members of each class. Here, we have to mention that in this classification scheme, the classes are usually termed families, but we chose as before [34,35] to reserve the term family for a lower level of classification. Class A of GPCRs (rhodopsin-like GPCRs) contains the majority of GPCRs, including receptors for structurally diverse ligands (biogenic amines, nucleotides, peptides, glycoprotein hormones etc). Class B (secretin-like GPCRs) contains purely peptide receptors, whereas class C (metabotropic glutamate family receptors) contains metabotropic glutamate and GABA-B receptors and some taste receptors. Class D contains the fungal pheromone receptors, class E contains the cAMP receptors of Dictyostelium and last is the Frizzled/Smoothened class. There are also a number of putative classes of newly discovered GPCRs, whose nomenclature has not been accepted yet from the scientific community. Further details for this higher level of classification can be found in [10,23,36] and in the references therein.

**Figure 2:**
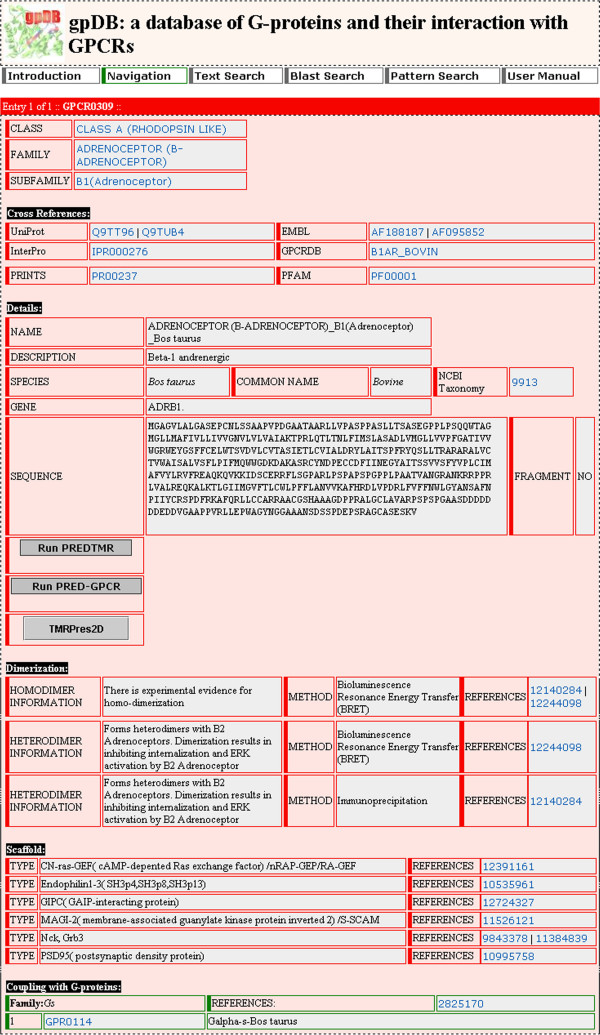
**A sample entry **Detailed view of an entry of the database. The user may observe, the classification of the protein, the available cross-references, the sequence, etc. At the bottom of the page, there is information concerning the coupling specificity of the particular protein; following any of the links, the user will be re-directed to the respective detailed view of the corresponding entry.

We further classified GPCRs into 64 different families and each family is further subdivided into different subfamilies, based mainly on TIPS classification scheme that takes into account the native ligand(s) that binds to a particular GPCR. Currently, information on coupling specificity is available only for GPCRs, belonging to the classes A, B, C, D, E and Frizzled/Smoothened, thus only GPCRs belonging to these classes are deposited in the database. A sample entry of the database is shown in Figure 2.

## Utility

**Figure 3:**
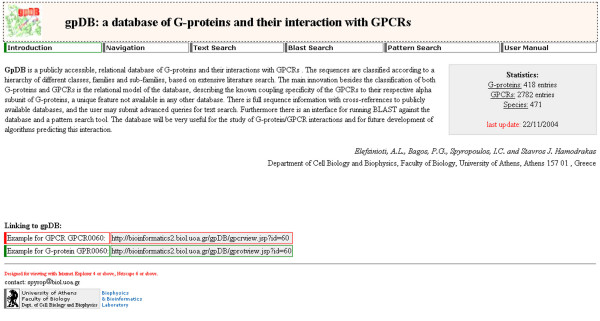
**The Home page of the database **A snapshot of the database's homepage. The main menu is shown with the available links for the tools, and the user's manual.

The application possesses a user-friendly environment, through which, the user may retrieve the necessary information, find available resources and cross-references and perform additional tasks such as running predictive algorithms, performing alignments, etc. In the main page of gpDB the user may find links for the following tools: Navigation, Text Search, BLAST Search, Pattern Search. There is also an extensive user's manual page, describing in detail the available tools (Figure 3). In summary, the available tools are summarised and described below.

### Navigation tool

Through the navigation tool, the user has the ability to browse the database following the hierarchy (Figure 1). The navigation can be performed on either the GPCR or the G PROTEIN hierarchy. Following the link of GPCRs, the user may be navigated through: GPCR CLASSES, GPCR FAMILIES, GPCR SUB-FAMILIES and individual RECEPTORS.

Alternatively, following the link of G PROTEINS, the user may browse through: G PROTEIN CLASSES, G PROTEIN FAMILIES, G PROTEIN SUB-FAMILIES, G PROTEIN TYPES and finally to individual G proteins. At each point, the user may navigate up or down the hierarchy tree. Finally, the user may obtain a detailed view of a particular GPCR or G protein (See Entry description).

### Text search tool

In the Text Search area, the user can search for any text in the fields of his/her preference. The user can enter any word in one or more of the available boxes under the name: 'Protein Name', 'Species', 'Description', 'Gene Name' and 'Cross-References'. Advanced queries can be performed using parentheses, and logical operators such as AND, OR, NOT, AND NOT as described in the documentation. Expressions in separate search fields are combined with the AND operator, so every entry of the result set will satisfy the expressions of all the search fields the user has chosen. The user has the option to choose whether the query will be performed against the GPCRs or the G proteins included in the database.

### BLAST tool

With the BLAST search tool [37], the user may submit a sequence and search the database for finding homologues. The user has the option to choose whether to perform the BLAST search against GPCRs sequences or G proteins sequences or both. The output of the BLAST query consists of a list of sequences in the database having significant E-values in a local pairwise alignment, ranked by statistical significance. Selecting a particular hit, the user may visualize the local alignment, and from there, may retrieve the detailed view of the entry corresponding to the particular target sequence.

### Pattern search tool

Using the Pattern Search tool (a home made tool), the user may perform searches for finding specific patterns in protein sequences of the database. The user, once again, has the option to choose whether to perform the Pattern search against the GPCR sequences or the G proteins sequences. The input of the Pattern Search tool could be either a standard regular expression pattern, or a pattern following the PROSITE [38] syntax. For example, the regular expression pattern:

DRY. [AGS].{3, 6}A

taken from the work of Moller and co-workers [21], that was shown to occur more frequently to the 2^nd ^intracellular loop of the Gi/o coupled GPCRs, has the simple interpretation, that we must have the consecutive residues Aspartate, Arginine Tyrosine (DRY), followed by any single residue(.), followed by one only of the following residues Alanine (A), Glycine (G) or Serine (S), followed by 3 to 6 residues of any type, ending up to an Alanine (A). We have collected, the 40 most discriminative patterns for each one of the three classes of coupling specificity, reported in [21] (found at ), and the user has the option, to use them in order to perform searches against the database.

The output of the Pattern search application consists of a list of the sequences matching the particular pattern. Following the appropriate links the user may retrieve the detailed view of the target sequence(s).

### Other tools

Furthermore, from the detailed view of an entry the user has the option to perform some additional tasks. These include, running PRED-TMR [39], PRED-GPCR [34,35] and TMRPres2D [40]. The aforementioned tools, will be extremely useful when it comes to GPCR sequences, for which the user may obtain predictions regarding the transmembrane segments, the family classification and the visual representation, respectively.

## Discussion

The database that we present here has some innovative and unique features not available in any other publicly accessible resource. The relational scheme, on which the database is organised, is especially designed to capture the coupling preferences of G proteins to GPCRs according to the reported data in the scientific literature. General sequence databases, such as Uniprot, do not include fields showing the coupling preference of GPCRs, but rather contain such information (if they do) in the free-text field of FUNCTION. Other specialised databases already exist focusing only in specific groups of receptors. For example GPCRDB [23], is the main publicly available resource for the classification of GPCRs. Other such approaches are the RTKdb [24], focusing on information of tyrosine protein kinase receptors and the Ligand-Gated Ion Channel Database, focusing on the Ligand-Gated Ion Channel receptors [25]. The database presented here however, not only combines information for both G proteins and GPCRs, but also includes information regarding their coupling specificity, the known effector molecules on which G proteins act, the accessory proteins interacting with GPCRs and information about the dimerization of GPCRs, all accompanied by links to original research articles from which the information was derived, features that are not available in any other publicly accessible resource. We have to note here, that gpDB does not aim at being a universal resource for GPCRs. A simple comparison with GPCRDB will show that the number of sequences included there, is at least two-three times larger than the sequences deposited in gpDB. This discrepancy arises from the fact that we do not report GPCRs, belonging to families, of which not a single member possesses a known coupling preference to G proteins. Thus, this database will be acting complementary to the existing databases regarding GPCRs, and interaction such as cross-referencing, will be useful.

The database provides a starting point for the development of algorithms predicting the coupling specificity of GPCRs to G proteins, an issue addressed already in the past by some teams [21,41], but with moderate success. This database consists of a larger, and well-organised dataset, on which we may build and test more effectively, such predictive algorithms. The database will be updated on a regular (yearly) basis, as new information emerges from genome sequencing projects, and verified experimentally. Also we plan to enrich the database in various ways, for instance developing methods for predicting the coupling specificity, and visualising, if possible, the potential interaction. Other possible additions, would be the the update of the relational scheme of the database in order to allow for dimeric receptors, for which information is already available and described in the database, or for hetero-trimeric G proteins, in case where information on specific subunit composition emerges. Furthermore, sequence information on the G proteins' effectors and the GPCRs' accessory proteins, could be combined in order to develop a fully automated computational resource for the study of protein-protein interactions in the cell membrane, that could describe the signal transduction to the interior of the cell.

The information, which the database comprises of, is essentially information regarding protein-protein interactions, which in turn, may be utilised in various ways. Currently, the most informative publicly available general purpose resource, concerning protein-protein interaction data, is the Database of Interacting Proteins [42]. Protein-protein interaction data arising either from databases, or from predictive algorithms may provide useful insight on the study of protein-protein interactions [43], but also may enable better functional annotation of proteins in the genomic context [44]. In particular, this kind of information may help in the construction of protein interaction networks, applied in genomic context [45].

## Conclusions

We present here a relational database, gpDB, summarizing the existing publicly available information regarding G proteins and their interactions with GPCRs. This database fills a gap in the already available resources, regarding GPCRs, and maintains an excellent functionality and interconnectivity with the publicly available databases and web-tools. The database is unique, since no other such database already exists, and will be useful for both molecular biologists conducting experiments, but also for bioinformaticians that manage large amount of data, building algorithms and performing functional classification of proteins in the genomic context.

## Availability and requirements

The gpDB is freely available for academic users at . Non-academics should contact Prof S. J. Hamodrakas at shamodr@cc.uoa.gr to obtain a license. All comments, suggestions, corrections and additions, should be sent to biodb@biol.uoa.gr.

## Authors' contributions

ALE carried out the data collection and annotation, classified the G proteins and drafted the manuscript, PGB designed the relational scheme of the database, classified GPCRs and drafted the manuscript, ICS implemented the SQL database and the web-server pages and SJH coordinated and supervised the whole project. All authors read and approved the final manuscript.
